# Effect of the frequently used antiepileptic drugs carbamazepine, gabapentin, and pregabalin on the pharmacokinetics of edoxaban and other oral factor xa inhibitors in healthy volunteers

**DOI:** 10.3389/fphar.2025.1542063

**Published:** 2025-04-11

**Authors:** Alexander Lenard, Simon A. Hermann, Felicitas Stoll, Juergen Burhenne, Kathrin I. Foerster, David Czock, Gerd Mikus, Andreas D. Meid, Walter E. Haefeli, Antje Blank

**Affiliations:** Internal Medicine IX, Department of Clinical Pharmacology and Pharmacoepidemiology, Heidelberg University, Medical Faculty Heidelberg/Heidelberg University Hospital, Heidelberg, Germany

**Keywords:** factor Xa inhibitors, edoxaban, carbamazepine, gabapentin, pregabalin, drug-drug interaction, microdose, healthy volunteers

## Abstract

**Purpose:**

Pregabalin, gabapentin, and carbamazepine, a potent inducer of cytochrome P450 (CYP) 3A4 and P-glycoprotein, are frequently used antiepileptic drugs that are often administered together with factor Xa inhibitors (FXaI). We aimed to investigate whether potentially clinically relevant drug-drug interactions occur with these combinations.

**Methods:**

In an open-label fixed-sequence trial in 36 healthy volunteers, we evaluated the pharmacokinetics of 60 mg edoxaban and of a microdosed FXaI cocktail (25 µg apixaban, 50 µg edoxaban, and 25 µg rivaroxaban) before and during treatment with carbamazepine (12 evaluable volunteers, individually dosed to therapeutic concentrations), gabapentin (11 volunteers, titrated to 3 × 400 mg/d), and pregabalin (12 volunteers, titrated to 2 × 300 mg/d). The antiepileptics were dosed to steady-state and the CYP3A activity was evaluated by assessing the pharmacokinetics of microdosed midazolam (30 µg).

**Results:**

Carbamazepine reduced the area under the plasma concentration-time curve ( ${\text{AUC}}_{\infty }$ ) of 60 mg edoxaban by a factor of 0.48 (geometric mean ratio (GMR) with 90% CI (0.41–0.56); p < 0.0001) and C_max_ by a factor of 0.47 (0.34–0.66) and reduced the exposure of the edoxaban metabolite M-4 to a similar extent. Carbamazepine also decreased the exposure ( ${\text{AUC}}_{\infty }$ ) of microdosed apixaban, edoxaban, and rivaroxaban by a factor of 0.66, 0.59, and 0.56, respectively. Gabapentin and pregabalin did neither affect the exposure of 60 mg edoxaban nor the exposure of any microdosed FXaI.

**Conclusion:**

Carbamazepine decreased FXaI exposure to a clinically relevant extent and dose adjustment may be required to maintain an adequate anticoagulant effect, whereas gabapentin and pregabalin do not require dose adjustment of FXaI.

## 1 Introduction

Direct-acting oral factor Xa inhibitors (FXaI) have achieved high prescription rates since their approval. They are at least as effective as vitamin K antagonists, have a favourable safety profile, an easy-to-follow fixed dosing regimen, and do not require routine monitoring (Giugliano et al., 2013; Granger et al., 2011; Hori et al., 2012; Hylek et al., 2014; Patel et al., 2011; Mao et al., 2014). Due to these favourable characteristics, FXaI are increasingly prescribed to the general population, especially to elderly and also to patients with chronic comorbidities such as neuropathic pain syndromes and seizures. After a stroke, patients often experience post-stroke pain syndromes (Paolucci et al., 2016) and also seizures (Galovic et al., 2021), which is why combination therapies of antiepileptic drugs (AED) and oral anticoagulants are common in these patients.

The anticoagulant effect of FXaI occurs rapidly and is concentration-dependent. As the differentiated dosing instructions for rivaroxaban show, even small dose changes that lead to correspondingly small changes in exposure play a major role in the efficacy and safety of this therapy. Several meta-analyses have revealed an association with an increased risk of all-cause mortality (Shen et al., 2023; Kong et al., 2021; Liu et al., 2021; Zhang et al., 2021) with underdosing, but a more precise understanding of the exact risks associated with off-label underdosing of FXaI is still lacking (Ohno et al., 2021). Increased risks of stroke (Kong et al., 2021; Liu et al., 2021; Zhang et al., 2021; Gronich et al., 2021), cardiovascular hospitalisation (Steinberg et al., 2016), and systemic embolism (Kong et al., 2021; Liu et al., 2021; Zhang et al., 2021; Gronich et al., 2021; Candeloro et al., 2022) have been reported as well as a significantly increased risk for adverse events (Steinberg et al., 2016). In contrast, a subgroup analysis of a Japanese population showed no significant association between any safety parameter and underdosing of FXaI (Murata et al., 2019). Because body weight and body mass index are significant risk factors for both stroke and death (Steinberg et al., 2016; Murata et al., 2019; Nielsen et al., 2017), these results can be confounded by these metrics. Accordingly, the recommended standard dose of rivaroxaban in Japan is 15 mg instead of the usual 20 mg (Murata et al., 2019). Therefore, changes in dose or clearance (CL) immediately translate into exposure and effect changes, and risks can arise from any pharmacokinetic drug-drug interaction (DDI).

Carbamazepine is a known substrate and inducer of CYP3A4 and P-glycoprotein (P-gp, ABCB1) with a relevant auto-induction of its own metabolism (Bertilsson et al., 1980). In patients, only few cases of combinations of enzyme-inducing drugs with direct oral anticoagulants have been reported and their results are heterogenous. As an example, subtherapeutic plasma concentrations and a transient ischemic attack were reported in a patient taking apixaban together with carbamazepine (Di Gennaro et al., 2019). In this single case, therapeutic plasma exposures were subsequently achieved with edoxaban with continued carbamazepine co-medication, but as there is no baseline value for comparison, a DDI between edoxaban and carbamazepine cannot be excluded. In contrast, in another case, discontinuation of carbamazepine had no effect on apixaban trough concentrations (Evanger et al., 2017). According to the summary of product characteristics, the combined use of apixaban with inducers of both CYP3A4 and P-gp such as carbamazepine can lead to a clinically relevant reduction of ∼50% in apixaban exposure (Eliquis, 2024). Indeed, in a large fraction of hospitalized patients (although by no means all) who received enzyme-inducing drugs together with apixaban, the exposure was substantially below the expected range (Perlman et al., 2019; Sennesael et al., 2021). Treatment failure has also been observed during co-administration of the enzyme-inducing phenobarbital with rivaroxaban or apixaban (Dagan et al., 2018). Furthermore, population-based and cohort studies found increased risks of ischemic stroke and other thromboembolic events among patients who were simultaneously treated with AED and FXaI (Ip et al., 2022; Giustozzi et al., 2021).

The pharmacokinetic properties, in particular the CL, differ between the marketed FXaI and depend on the individual characteristics of the transport, metabolic, and excretion pathways involved. Drug transporters such as P-gp, phase-I metabolic enzymes (CYP3A, CYP2J2, and carboxylesterase 1 (CES1)), and phase-II enzymes (e.g., uridine 5′-diphospho -glucuronosyltransferases) play a variable role, as does renal elimination, which accounts for 25%–50% of FXaI CL (Lixiana, 2015; Eliquis, 2024; Xarelto, 2024; Foerster et al., 2020). These differences in the CL pathways determine the DDI potential with modulators of the corresponding CL pathways and make apixaban and rivaroxaban sensitive to CYP modulators, whereas edoxaban is not substantially metabolized by CYP enzymes.

Edoxaban elimination mainly depends on P-gp, polymorphic CES1, and CYP3A4. It is metabolized by CES1 to its active edoxaban metabolite M-4 (M-4). Further metabolisation pathways of M-4 are not yet fully understood but it is substrate to the hepatic liver-specific organic anion transporter 1 (OATP1B1). Apixaban as well as rivaroxaban are biotransformed mainly via CYP3A4. Accordingly, apixaban and rivaroxaban have a cautionary statement on the use of CYP3A4 inducers in their summary of product characteristics (Foerster et al., 2020).

The AED on the market also have different perpetrator properties. Pregabalin and gabapentin have no enzyme-inducing or enzyme-inhibiting effects and their risk for DDI is comparatively low (Summary of product characteristics, 2018; Bockbrader et al., 2010; Wang et al., 2010). They are eliminated by renal excretion, particularly via active tubular secretion involving the OCTN1 (Neurontin, 2018; Bockbrader et al., 2010; Urban et al., 2008; Solvobiotech, 2018). On the other hand, carbamazepine is known for its induction of cytochrome enzymes such as CYP1A2, CYP2C9, and CYP3A4, as the efflux transporter P-gp, but the magnitude of this effect on FXaI exposure has not been well studied and clinical conclusions could be better drawn if the extent of DDI is known and if well-designed physiologically based pharmacokinetic modelling would be available (Candeloro et al., 2022; Puma et al., 2020).

Pharmacokinetic DDI are not class phenomena and need to be examined case by case. The comparability of DDI data for FXaI can be improved by administering different FXaI to the same individual. However, simultaneous administration of therapeutic doses poses a risk and consecutive administration requires long washout periods, which prolongs trial duration. Both challenges can be overcome by using a microdose approach for DDI trials, where more than one FXaI can be administered as a microdose with negligible effects on coagulation (Hohmann et al., 2015; Lenard et al., 2024; Mikus et al., 2019; Mikus et al., 2020). In addition, simultaneous administration to one individual reduces intra-individual and inter-individual variability and allows for a safe and timely study design (Mikus et al., 2019).

We used this approach to assess the effect of therapeutic doses at steady-state of carbamazepine, gabapentin, and pregabalin on the pharmacokinetics of a single therapeutic dose of edoxaban and a microdose cocktail of three FXaI (apixaban, edoxaban, rivaroxaban) and evaluated the utility of the microdosed FXaI cocktail for assessing pharmacokinetic DDI.

## 2 Materials and methods

### 2.1 Ethical approval

The trial protocol was approved by the competent authority (BfArM, Bonn, Germany, #4043377), received a positive vote from the responsible Ethics Committee of the Medical Faculty of Heidelberg University, Germany (AFmo-144/2019), and was registered in the EudraCT database (EudraCT 2018-002490-22). This investigator-initiated, monocentre phase-I trial adhered to the Declaration of Helsinki, the principles of good clinical practice, and all pertinent legal requirements in Germany. The trial took place at the DIN EN ISO 9001-certified pharmacological early clinical trial unit (KliPS) of Internal Medicine IX, Department of Clinical Pharmacology and Pharmacoepidemiology at Heidelberg University Hospital.

### 2.2 Trial population and design

Healthy volunteers aged 18–65 years were eligible to participate in the trial after giving written informed consent. Inclusion and exclusion criteria ensured that the participants were in good health and had no relevant medical history or relevant findings in laboratory values, electrocardiogram, and physical examination. Volunteers were also required to adhere to strict pregnancy prevention measures.

**FIGURE 1:**
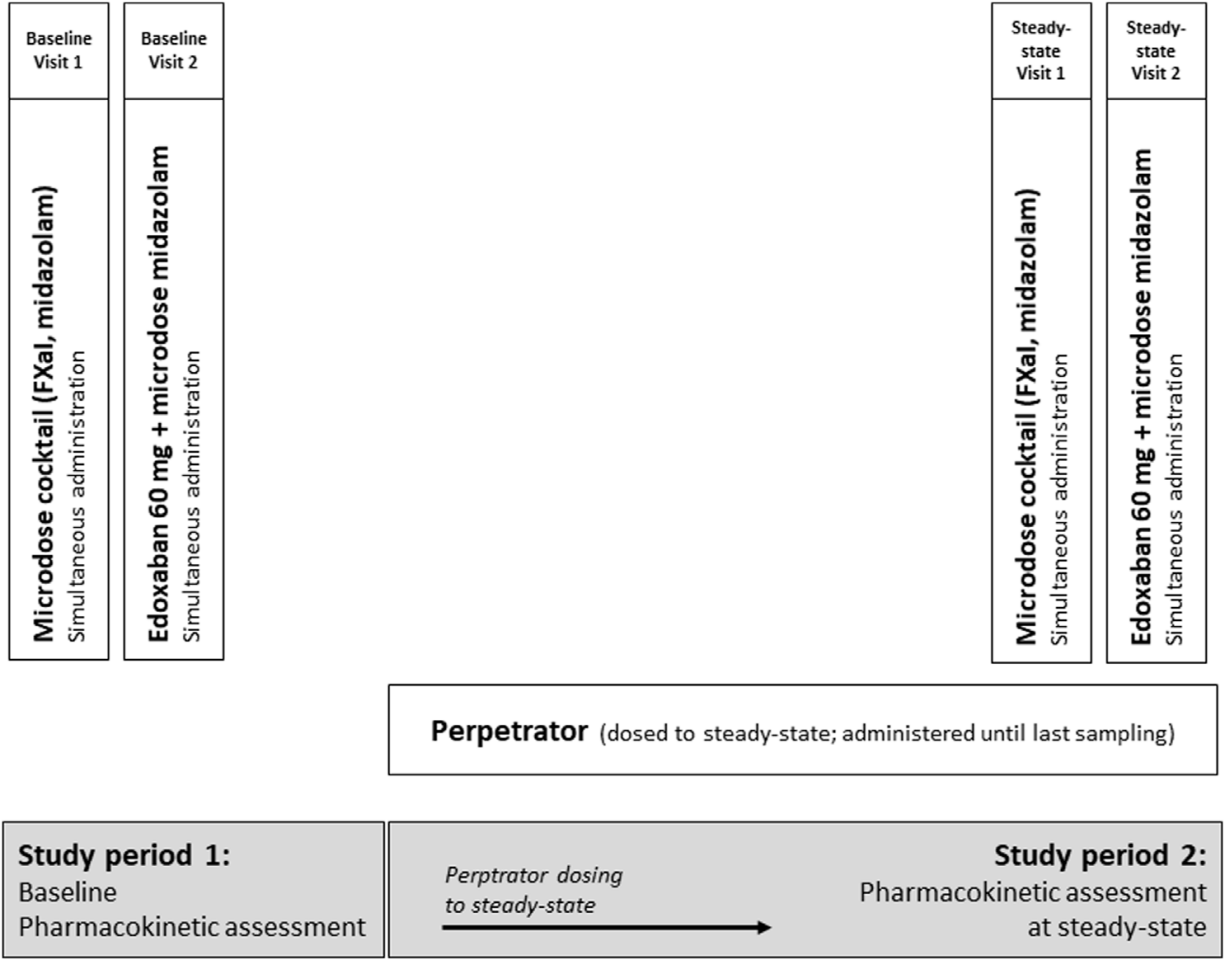
Trial design assessing the impact of carbamazepine, pregabalin, and gabapentin (therapeutic doses, all dosed to steady-state) on the pharmacokinetics of a single oral dose of 60 mg edoxaban and on a microdosed factor Xa inhibitor cocktail, containing 25 μg apixaban, 50 μg edoxaban, and 25 μg rivaroxaban in healthy volunteers. In addition, the effect of perpetrators on microdosed midazolam (30 μg) as marker for CYP3A4 was assessed.

The trial was an open-label, two-period DDI trial with three cohorts of healthy volunteers to assess the impact of carbamazepine, pregabalin, and gabapentin on the pharmacokinetics of 60 mg edoxaban (primary endpoint), given as a tablet, and on the pharmacokinetics of apixaban (25 µg), edoxaban (50 µg), and rivaroxaban (25 µg) administered as an oral microdose cocktail (secondary endpoints, (Mikus et al., 2019)). We simultaneously quantified the effect on CYP3A activity with an oral midazolam microdose (30 µg) (Figure 1).

Carbamazepine, gabapentin, and pregabalin were dosed to steady-state during a treatment period of 21 d for carbamazepine, 13 d for gabapentin, and 21 d for pregabalin, followed by the evaluation of their perpetrator effects. Steady-state was ensured until the last blood sample was drawn. Carbamazepine was titrated to a maximum dose of 200 mg twice a day, if possible. If this dose could not be reached due to adverse events, carbamazepine was titrated to therapeutic plasma concentrations (5–10 μg/mL), with the lower range chosen to ensure the safety of the participants. Gabapentin was administered at a dose of 400 mg three times a day and pregabalin at a dose of 300 mg twice a day. Single-dose pharmacokinetics of the microdosed FXaI were assessed at baseline and at the end of the above mentioned treatment period, at steady-state of the perpetrator. The therapeutic 60-mg dose of edoxaban edoxaban was tested thereafter on the following day, both at baseline and at steady-state. All microdosed FXaI and the 60 mg dose of edoxaban were administered under supervision and at least 60 min after administration of the perpetrator. The midazolam microdose was administered as an oral solution simultaneously with administering the FXaI (Katzenmaier et al., 2010; Katzenmaier et al., 2011).

### 2.3 Quantification of FXaI, edoxaban metabolite M-4, and midazolam

Venous blood samples were drawn before and 0.25, 0.5, 0.75, 1, 1.5, 2, 2.5, 3, 3.5, 4, 5, 6, 8, 10, 12, 24 (last sampling time after microdoses), and 48 h (edoxaban 60 mg) after drug administration. We assessed the pharmacokinetics of midazolam using a limited sampling strategy with sampling at 2, 2.5, 3, and 4 h post dose (Katzenmaier et al., 2010; Katzenmaier et al., 2011). All collected samples were processed within 20 min and plasma was stored at ≤ −20°C until analysis. Coagulation markers (activated partial thromboplastin time and international normalized ratio) were measured at expected FXaI peak plasma concentrations (C_max_, 3 h post dose) in the accredited central laboratory of Heidelberg University Hospital.

Plasma concentrations of apixaban, edoxaban, edoxaban metabolite 4 (M-4), midazolam, and rivaroxaban were measured with ultra-sensitive, ultra-performance liquid chromatography tandem mass spectrometry (UPLC-MS/MS) methods utilising accuracy and precision values of less than or equal to ±15% as described previously (Burhenne et al., 2012; Foerster et al., 2018). All methods were validated according to pertinent guidelines (U.S. Food and Drug Administration, 2023; European medicines agency, 2022). In brief, apixaban, edoxaban, edoxaban M-4, and rivaroxaban were extracted from plasma using Waters Oasis HLB Prime µElution 96-well plates by solid phase extraction (SPE). The resulting extracts were gradient chromatographed on a Waters BEH C18 column using water and acetonitrile. Detection was performed on a Waters Xevo TQ-S system (micro dose) or on a Waters TQD system (high dose) in electrospray positive mode and multiple reaction monitoring. The applied lower limits of quantification were 2.5 pg/mL for all microdosed FXaI, and 1 ng/mL for regular doses of edoxaban.

Midazolam was extracted from plasma using Waters Oasis MCX µElution 96-well plates by SPE and the resulting extracts were gradient chromatographed on a Waters BEH C18 column using water and acetonitrile too. Detection was also performed on a Waters Xevo TQ-S system in electrospray positive mode and multiple reaction monitoring. The applied lower limit of quantification was 1 pg/mL for the microdosed midazolam.

### 2.4 Pharmacokinetic and statistical analysis

Non-compartmental analyses were conducted to assess the pharmacokinetics of the FXaI and midazolam utilizing Phoenix WinNonlin 8.3 (Certara, Inc., Princeton, NJ, United States). C_max_ and the time to reach C_max_ (t_max_) were directly obtained from the concentration data. The area under the concentration-time curve from 0 to infinity ( ${\text{AUC}}_{\infty }$ ) was determined using the log-linear trapezoidal rule with extrapolation to infinity. The half-life (t_1/2_) was calculated as $\frac{\ln\left(2\right)}{\lambda z}$ , with the elimination rate constant λ_z_ being estimated utilizing log-linear regression of the elimination phase. Apparent oral CL (CL/F) and volume of distribution (V_z_/F) were calculated using standard equations for non-compartmental analysis. CYP3A4 activity was assessed using the estimated partial metabolic CL (eCL_met_) as described earlier (Katzenmaier et al., 2010; Katzenmaier et al., 2011; Hohmann et al., 2015).

Parameters are presented as geometric means (GM) with 95% confidence interval (CI). Exposure changes were evaluated by using the geometric mean ratio (GMR) of ${\text{AUC}}_{\infty }$ and C_max_ at baseline and during the steady-state of the perpetrator (carbamazepine, gabapentin, pregabalin) with 90% CI. The correlations were calculated using the Pearson correlation, reporting *R* ^2^ with a two-sided 95% CI. The AUC_2-4_ and eCL_met_ of midazolam were expressed as geometric means (GM) with 95% confidence interval and were tested by repeated-measures analysis of variance after logarithmic transformation using assessments at multiple time points. Statistical analyses and graphical displays were carried out using Prism 9.5.1 (GraphPad Software Inc., La Jolla, CA, United States). A p-value < 0.05 was considered significant.

## 3 Results

After providing the participants with comprehensive information and obtaining their written informed consent, we enrolled 36 healthy volunteers (22 females, 14 males), aged 21–63 years (median 26), with a mean body mass index of 23.97 kg/m^2^ (± standard deviation (SD) 3.79) in the three cohorts of this trial. The cohorts consisted of 13, 11, and 12 volunteers to evaluate the effects of carbamazepine, gabapentin, and pregabalin, respectively. One participant of the carbamazepine cohort did not complete the trial due to an adverse event (hypersensitivity with fever and liver enzyme elevation). Therefore, the carbamazepine cohort consisted of 12 evaluable individuals.

### 3.1 Effect of carbamazepine on FXaI pharmacokinetics

**FIGURE 2:**
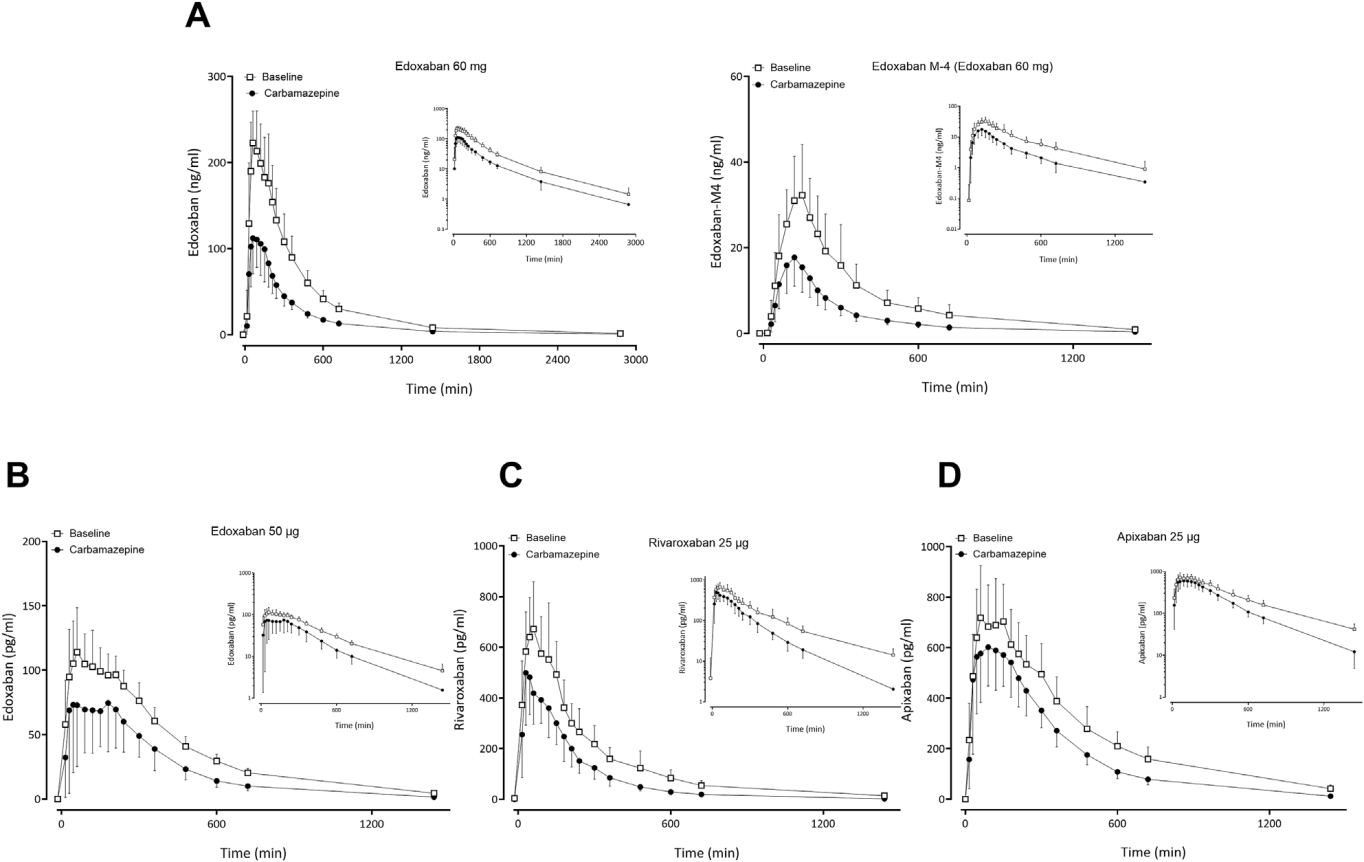
Mean (± standard deviation) plasma concentration-time profiles of factor Xa inhibitors after administration of single oral doses before carbamazepine (open squares) and at carbamazepine steady-state (solid circles) to 12 healthy volunteers: **(A)** edoxaban 60 mg and edoxaban M-4 metabolite, **(B)** apixaban 25 μg, **(C)** edoxaban 50 µg, **(D)** rivaroxaban 25 µg.

At carbamazepine steady-state (21 d ± 0 d), full induction by carbamazepine for all participants was assumed. Compared to baseline, the GMR of ${\text{AUC}}_{\infty }$ and C_max_ of 60 mg edoxaban were 0.48 and 0.47, respectively. The corresponding values of the M-4 metabolite were 0.48 ( ${\text{AUC}}_{\infty }$ ) and 0.51 (C_max_) (Table 1; Figure 2;Supplementary Figure S1), and the CL/F values of edoxaban and M-4 were both increased 2.1-fold (Supplementary Table S1). Carbamazepine did not change the molar metabolic ratio of edoxaban to its metabolite edoxaban M-4 (GMR 0.99; 90% CI: 0.85–1.15). Accordingly, the intraindividual correlation of the metabolic ratio at baseline and at carbamazepine steady-state was significant (Pearson *R* ^2^ = 0.66, p = 0.02). Carbamazepine also reduced ${\text{AUC}}_{\infty }$ and C_max_ of all three FXaI administered as a microdose cocktail (Table 1; Figure 2; Supplementary Figure S2; Supplementary Figure S3; Supplementary Figure S4) and correspondingly increased their CL/F (Supplementary Table S1).

**TABLE 1:** Pharmacokinetic parameters of factor Xa inhibitors at baseline and at carbamazepine steady-state (N = 12 healthy volunteers).

Drug	Pharmacokinetic variable	Baseline		During carbamazepine		Change*	
		*GM (95 % CI)*		*GM (95 % CI)*		*GMR (90 % CI)*	
Edoxaban (60 mg)	AUC_∞_ (min*µg/mL)	86.6	(70.9−106)	41.2	(34.9−48.7)	0.48	(0.41−0.56)
	C_max_ (ng/mL)	267	(220−323)	127	(90−179)	0.47	(0.34−0.66)
Edoxaban M-4	AUC_∞_ (min*µg/mL)	9.97	(6.93−14.3)	4.78	(3.52−6.48)	0.48	(0.39−0.58)
	C_max_ (ng/mL)	31	(22−44)	16	(11−23)	0.51	(0.33−0.77)
Edoxaban/ edoxaban M-4	MR of AUCs (molar)	8.26	(6.5−10.4)	8.19	(6.4−10.4)	0.99	(0.85−1.15)
µ-edoxaban (50 µg)	AUC_∞_ (min*ng/mL)	53	(45−63)	31	(25−39)	0.59	(0.52−0.67)
	C_max_ (pg/mL)	119	(95−150)	83	(60−115)	0.70	(0.60−0.81)
µ-apixaban (25 µg)	AUC_∞_ (min*ng/mL)	354	(279−449)	235	(190−290)	0.66	(0.59−0.74)
	C_max_ (pg/mL)	719	(574−901)	634	(472−850)	0.88	(0.79−0.98)
µ-rivaroxaban (25 µg)	AUC_∞_ (min*ng/mL)	186	(143−242)	104	(82−131)	0.56	(0.49−0.64)
	C_max_ (pg/mL)	695	(551−878)	524	(401−686)	0.75	(0.66−0.86)

AUC_∞_, area under the plasma concentration-time curve extrapolated to infinity (extrapolated fraction < 19% for the administered FXaI, <38% for edoxaban M-4); CI, confidence interval; C_max_, peak plasma concentration; GM, geometric mean; GMR, geometric mean ratio; MR, metabolic ratio). *All statistical comparisons between the baseline value and the value under carbamazepine were statistically significant (p ≤ 0.005).

At baseline, the dose-normalized ${\text{AUC}}_{\infty }$ of the edoxaban microdose was slightly but significantly smaller than the ${\text{AUC}}_{\infty }$ of the therapeutic dose (dose-normalized GMR 0.74; 90% CI: 0.67–0.81, p = 0.0002), while at carbamazepine steady-state, there was no significant difference between the two groups (GMR 0.91; 90% CI: 0.77–1.09; p = 0.3, Supplementary Figure S5). The ratios of carbamazepine-mediated change in ${\text{AUC}}_{\infty }$ of the therapeutic dose were significantly larger than the ratios observed after administration of the microdose (Table 1; Figure 2, GMR of ratios 1.24; 1.0–1.52, p = 0.046). There was a significant intra-individual correlation between the ${\text{AUC}}_{\infty }$ of edoxaban 60 mg and edoxaban 50 μg at baseline (Pearson correlation coefficient *R* ^2^ = 0.64, two-sided p = 0.002) and during carbamazepine steady-state (*R* ^2^ = 0.35, p = 0.04). Additionally, there was a significant correlation between the C_max_ of edoxaban 60 mg and edoxaban 50 μg at baseline (Pearson correlation coefficient *R* ^2^ = 0.70, p = 0.01; Supplementary Figure S6). Changes in the AUC of the microdose during induction were not predictive of the changes observed with a therapeutic dose, as there was no correlation between the magnitude of intra-individual ${\text{AUC}}_{\infty }$ changes (Pearson correlation coefficient *R* ^2^ = 0.10, two-sided p = 0.3). In contrast to the microdosed FXaI, there was a significant correlation between the decrease of AUC of 60 mg edoxaban during induction and AUC of 60 mg edoxaban at baseline (*R* ^2^ = 0.4, p = 0.02; Supplementary Figure S7).

### 3.2 Effect of gabapentin and pregabalin on FXaI pharmacokinetics

**FIGURE 3:**
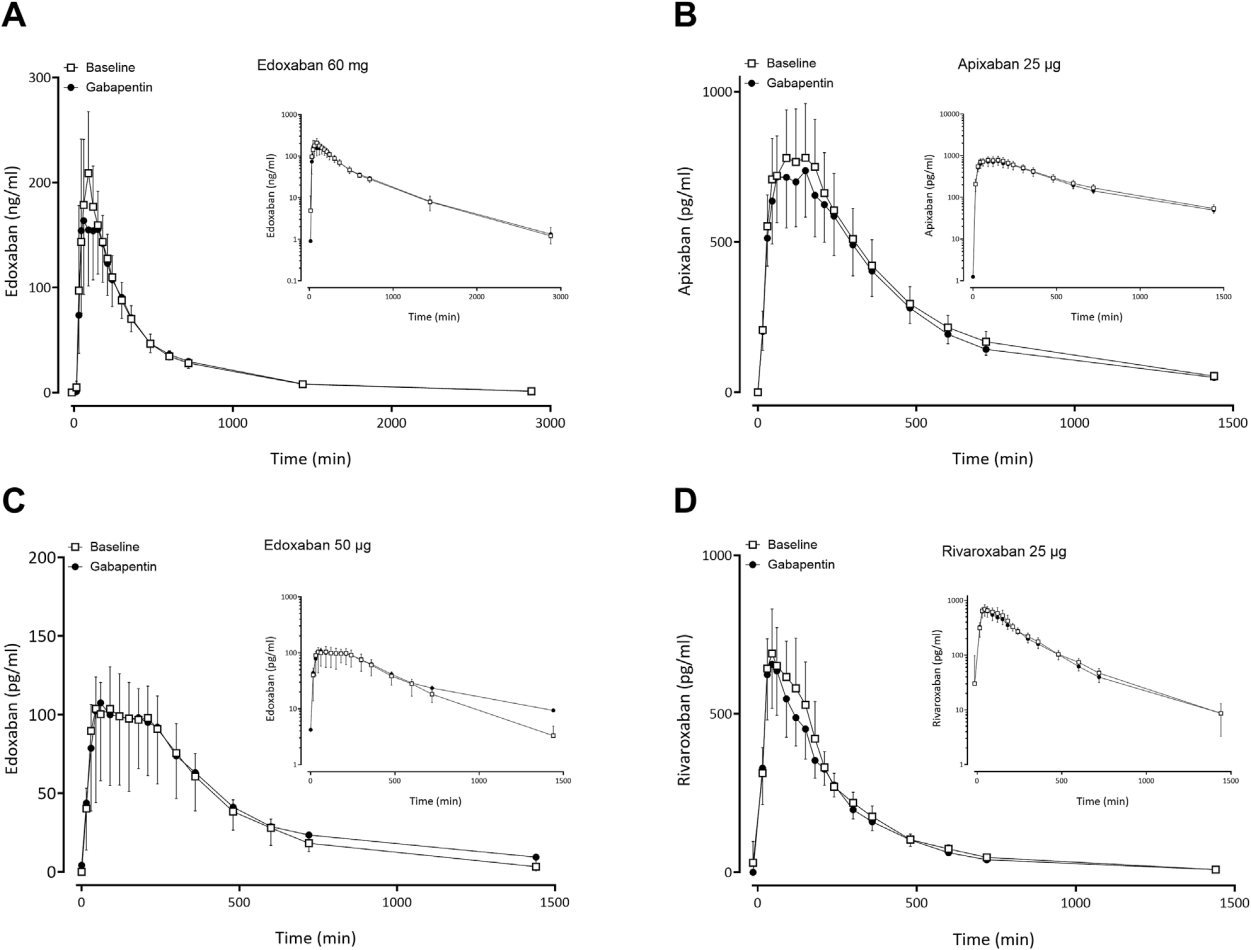
Mean (± standard deviation) plasma concentration-time profiles of factor Xa inhibitors after administration of single oral doses before gabapentin (open squares) and at gabapentin steady-state (solid circles) to 11 healthy volunteers: **(A)** edoxaban 60 mg, **(B)** apixaban 25 µg, **(C)** edoxaban 50 μg, **(D)** rivaroxaban 25 µg.

**FIGURE 4:**
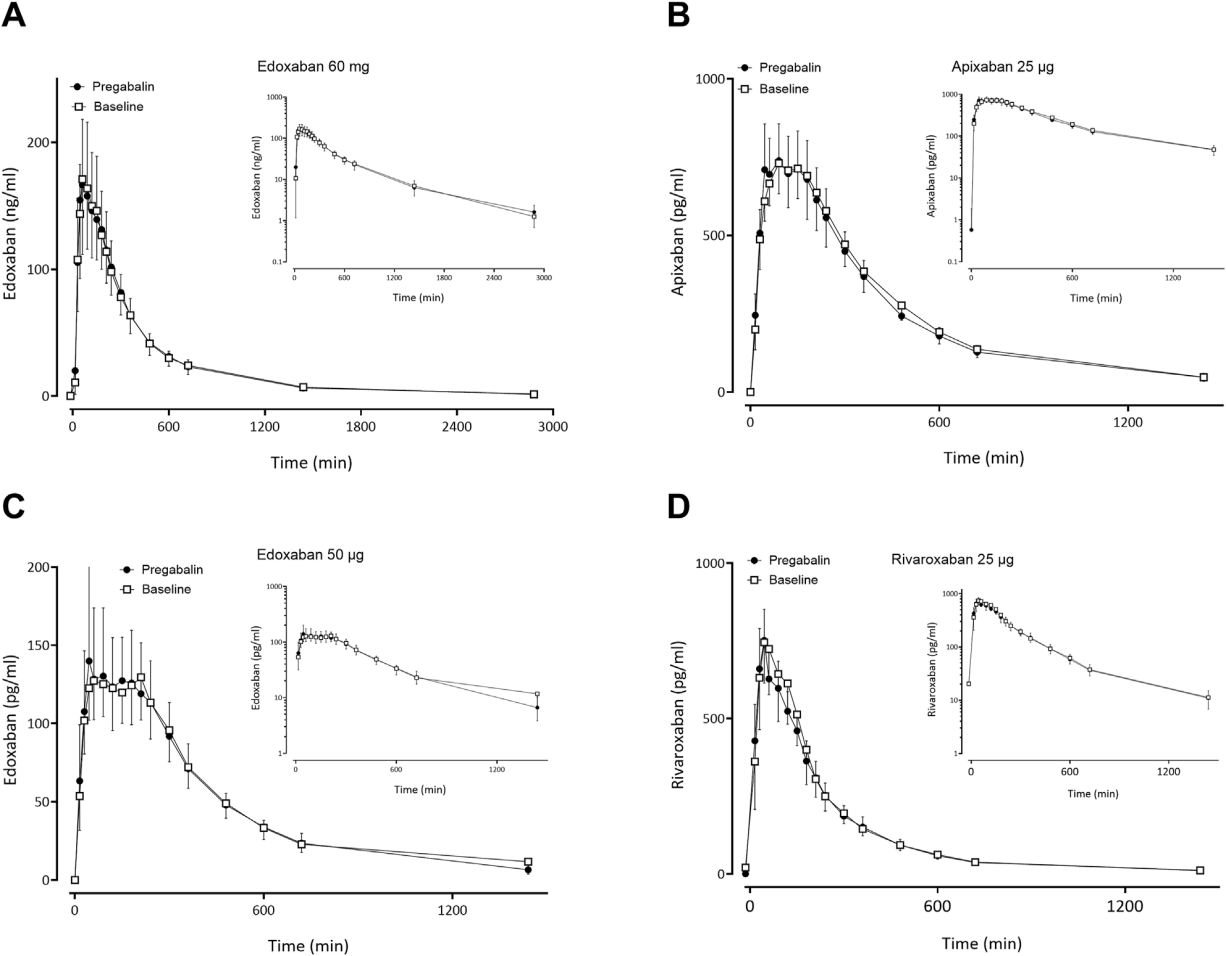
Mean (± standard deviation) plasma concentration-time profiles of factor Xa inhibitors after administration of single oral doses before pregabalin (open squares) and at pregabalin steady-state (solid circles) to 12 healthy volunteers: **(A)** edoxaban 60 mg, **(B)** apixaban 25 µg, **(C)** edoxaban 50 μg, **(D)** rivaroxaban 25 µg.

Gabapentin and pregabalin did not alter the exposure of a therapeutic 60 mg dose of edoxaban or FXaI administered as a microdose cocktail (Table 2; Table 3; Figures 3, 4).

**TABLE 2:** Pharmacokinetic parameters of factor Xa inhibitors at baseline and at gabapentin steady-state (N = 11 healthy volunteers).

Drug	Pharmacokinetic variable	Baseline		During gabapentin		Change*	
		*GM*	*(95 % CI)*	*GM*	*(95 % CI)*	*GMR*	*(90 % CI)*
Edoxaban (60 mg)	AUC_∞_ (min*µg/mL)	75	(65−88)	73	(61−87)	0.96	(0.78−1.2)
	C_max_ (ng/mL)	227	(170−305)	182	(129−256)	0.8	(0.53−1.21)
µ-edoxaban (50 µg)	AUC_∞_ (min*ng/mL)	50	(43−60)	56	(43−74)	0.91	(0.93−1.35)
	C_max_ (pg/mL)	112	(89−141)	118	(89−154)	1.05	(0.88−1.25)
µ-apixaban (25 µg)	AUC_∞_ (min*ng/mL)	417	(347−501)	389	(328−462)	0.93	(0.82−1.07)
	C_max_ (pg/mL)	822	(659−1,025)	775	(613−980)	0.94	(0.81−1.10)
µ-rivaroxaban (25 µg)	AUC_∞_ (min*ng/mL)	194	(167−224)	179	(157−205)	0.92	(0.84−1.02)
	C_max_ (pg/mL)	718	(595−550)	688	(550−861)	0.96	(0.82−1.12)

AUC_∞_, area under the plasma concentration-time curve extrapolated to infinity (extrapolated fraction < 15%); CI, confidence interval; C_max_, peak plasma concentration; GMR, geometric mean ratio. *None of the statistical comparisons between the baseline value and the value under gabapentin was statistically significant.

**TABLE 3:** Pharmacokinetic parameters of factor Xa inhibitors at baseline and at pregabalin steady-state (N = 12 healthy volunteers).

Drug	Pharmacokinetic variable	Before pregabalin		During pregabalin		Change*	
		*GM*	*(95 % CI)*	*GM*	*(95 % CI)*	*GMR*	*(90 % CI)*
Edoxaban (60 mg)	AUC_∞_ (min*µg/mL)	67	(56−80)	63	(46−88)	0.94	(0.78−1.15)
	C_max_ (ng/mL)	170	(124−234)	161	(98−263)	0.94	(0.65−1.37)
µ-edoxaban (50 µg)	AUC_∞_ (min*ng/mL)	71	(59−85)	67	(59−77)	0.95	(0.84−1.07)
	C_max_ (pg/mL)	148	(126−173)	150	(122−186)	1.02	(0.87−1.20)
µ-apixaban (25 µg)	AUC_∞_ (min*ng/mL)	372	(322−430)	364	(318−416)	0.98	(0.86−1.11)
	C_max_ (pg/mL)	782	(682−897)	775	(653−919)	0.99	(0.86−1.13)
µ-rivaroxaban (25 µg)	AUC_∞_ (min*ng/mL)	185	(151−227)	182	(158−208)	0.98	(0.83−1.15)
	C_max_ (pg/mL)	783	(643−954)	744	(644−859)	0.95	(0.79−1.13)

AUC_∞_, area under the plasma concentration-time curve extrapolated to infinity (the extrapolated fraction was <21%); CI, confidence interval; C_max_, peak plasma concentration; GMR, geometric mean ratio. *None of the statistical comparisons between the baseline value and the value under pregabalin were statistically significant.

### 3.3 Assessment of CYP3A activity

Co-administration of carbamazepine increased eCL_met_ 5.52-fold and decreased midazolam AUC_2-4_ by 82% (GMR 0.18; p < 0.0001) (Supplementary Table S2). The mean decrease of midazolam AUC_2-4_ was significantly larger than the mean decrease of the ${\text{AUC}}_{\infty }$ of individual FXaI (p < 0.001). To test whether the decrease in midazolam AUC_2-4_ as a marker of CYP3A4 induction was related to the decrease in ${\text{AUC}}_{\infty }$ of edoxaban 60 mg or M-4, we tested their correlations and found that they did not correlate with each other. There was a significant linear correlation between the extent of decrease (ratio) of midazolam and the microdose of edoxaban (*R* ^2^ = 0.70, p < 0.001; Supplementary Figure S8). Gabapentin and pregabalin did not alter midazolam pharmacokinetics (Supplementary Table S2).

### 3.4 Effect on coagulation

Given alone, the therapeutic edoxaban dose of 60 mg significantly increased activated partial thromboplastin time and international normalized ratio values 3 h after administration; these effects were significantly smaller at carbamazepine steady-state (Supplementary Table S3). The coagulation changes induced by edoxaban in therapeutic dose or microdose were not influenced by gabapentin or pregabalin (data not shown).

### 3.5 Safety

#### 3.5.1 Carbamazepine cohort

Overall, 29 adverse events (AE) occurred in 12 of 13 participants, 19 events were deemed possibly related to the trial medication. All AE were transient, none was serious, one was severe (fever in combination with liver enzyme elevation) and resulted in immediate carbamazepine discontinuation, withdrawal from the trial, and monitoring until symptoms completely resolved. The most frequent AE was nausea reported by four participants while taking carbamazepine. Other AE occurring more than once were fatigue (n = 3), abdominal pain (2), alanine aminotransferase elevation (2), headache (2), and dizziness (2), which all occurred during carbamazepine treatment.

#### 3.5.2 Gabapentin cohort and pregabalin cohort

Overall, 39 AE occurred in 11 of 11 participants in the gabapentin-cohort, 21 of which were deemed possibly related to the trial medication or procedures. All AE were transient, none was severe or serious, and none resulted in withdrawal from the trial. The most frequent AE was flu-like symptoms, reported by five participants while taking gabapentin. Other AE occurring more than once were headache (n = 4), fatigue and dizziness (3), creatine kinase elevation (2), euphoria (2), vertigo (2), and nausea (2), which all occurred during gabapentin treatment.

Overall, 37 AEs occurred in 11 of 12 participants in the pregabalin cohort, 25 of which were deemed possibly related to the trial. All AE were transient, none was severe or serious, and none resulted in withdrawal from the trial. The most frequent AE were coordination difficulties (n = 3), impaired vision (3), headache (3), vertigo (3), nausea (2), vomiting (2), flulike symptoms (2), fatigue (2), diarrhoea (2), restlessness (2), and somnolence (2), which all occurred during pregabalin treatment.

## 4 Discussion

### 4.1 Influence of carbamazepine on the pharmacokinetics of 60 mg edoxaban and the microdosed FXaI cocktail

The P-gp and CYP3A4 inducer carbamazepine decreased the AUC and C_max_ of a therapeutic edoxaban dose by about half and reduced the exposure parameters of the active metabolite M-4 in the same order of magnitude and direction. The most likely, primary mechanism for this is a decreased bioavailability due to induction of P-gp in the gut (Mikkaichi et al., 2014). The observation that both CL/F and Vz/F were approximately doubled, while t_1/2_ remained unchanged, suggests that the observed pharmacokinetic changes are mainly due to a substantial reduction in F. This is also confirmed by the fact that the metabolic ratio of edoxaban and M-4 is not affected by carbamazepine because M-4 is a product of CES1 which is not induced by carbamazepine.

Both, efficacy and safety of FXaI treatment, previously showed correlations to FXaI exposure (Shen et al., 2023; Kong et al., 2021; Liu et al., 2021; Zhang et al., 2021; Ohno et al., 2021; Gronich et al., 2021; Steinberg et al., 2016; Murata et al., 2019; Nielsen et al., 2017; Vasanthamohan et al., 2018; Herink et al., 2019). Retrospective evidence from cohort analyses indicated that co-medication leading to increased FXaI exposure is associated with major bleeding events as well as an increased risk of stroke and all-cause mortality (Shen et al., 2023; Kong et al., 2021; Liu et al., 2021; Zhang et al., 2021; Ohno et al., 2021; Gronich et al., 2021; Steinberg et al., 2016; Murata et al., 2019; Nielsen et al., 2017; Vasanthamohan et al., 2018; Holm et al., 2021; Harskamp et al., 2019; Li et al., 2020; Ray et al., 2024). Conversely, combinations that reduce exposure appear to increase the risk of thromboembolic events by 60% and more and increase the likelihood of strokes (Gronich et al., 2021; Holm et al., 2021). The co-administration of edoxaban with rifampin, a strong CYP3A and P-gp inducer, requires monitoring of anticoagulation according to the marketing authorisation (Lixiana, 2017), as AUC reductions of up to 35% occurred. In healthy volunteers, the exposure of edoxaban decreased during a 7-d rifampin treatment while the effects on the active metabolites M-4 and M-6 were opposite and the AUC of M-4 increased 2.86-fold and C_max_ 5.06-fold and its t_1/2_ decreased (Mendell et al., 2015). This obvious difference in the interaction pattern of rifampin compared to carbamazepine can be explained by the fact that M-4, but not edoxaban, is an OATP1B substrate (Mikkaichi et al., 2014) whose hepatic uptake is inhibited by rifampin but not by carbamazepine, which does not interact with OATP1B at therapeutic concentrations (Gui et al., 2008). M-4 and edoxaban have comparable anticoagulant effects (Brown, 2014). If the total anticoagulant exposure is calculated by adding up the molar concentrations of edoxaban and M-4, the interaction with rifampin leads to a reduction of only 17%, as M-4 is tripled at the same time. In contrast, the analogue calculation for carbamazepine leads to a 52% reduction in the anticoagulant effect, which effectively corresponds to a halving of the dose, a very significant change. Based on this, caution must be taken to avoid loss of effect of edoxaban under carbamazepine, strict observation of anticoagulation effects is recommended, and a dose increase should be considered.

As expected, gabapentin and pregabalin did not change the exposure of edoxaban, and these combinations can therefore be safely used in patients.

### 4.2 Evaluation of the potential DDI by using a microdosed cocktail

In separate trial arms, we quantified the effect of the three perpetrators carbamazepine, gabapentin, and pregabalin on a microdosed cocktail consisting of three simultaneously administered FXaI and were able to intra-individually compare the impact of each perpetrator on the FXaI. For gabapentin and pregabalin, the microdose data correctly predicted that there was no interaction with the regular edoxaban dose. The exposure changes during carbamazepine were significantly, albeit not substantially smaller with microdoses of edoxaban than the exposure reductions found with therapeutic doses of edoxaban (reduction of AUC to 59% versus 48%, respectively), indicating that the evaluation with microdoses slightly underestimated the magnitude of interaction in a therapeutic setting. While linear pharmacokinetics has been confirmed between 10 mg and 150 mg doses of edoxaban (Moon, 2019), linearity has not been thoroughly assessed for microdoses. It has been shown that metabolic and intestinal drug transport pathways for some drugs may be non-linear, which may explain why exposure after a microdose only approximately predicts the corresponding exposure after a therapeutic dose (Ieiri et al., 2012; Maeda et al., 2011). It remains to be fully understood why the decrease in midazolam AUC_2-4_ as a marker of CYP3A4 induction (ratio) did not correlate to the decrease in ${\text{AUC}}_{\infty }$ of edoxaban 60 mg or edoxaban M-4 (ratio), but there was a significant linear correlation between the ratio of midazolam AUC_2-4_ and microdosed edoxaban AUC. A possible explanation for this is as mentioned above that metabolic and intestinal drug transport pathways may be non-linear and therefore the correlation for the exposure after a microdose of edoxaban could not be confirmed with the therapeutic dose of edoxaban. However, in general the parameters and the perpetrator effects were in a similar range, and the difference was not large enough to draw different final conclusions from the two settings. Therefore, in the case of edoxaban, a microdose assessment of a transporter DDI is still conclusive and advantageous if DDI are to be evaluated in patients. We have recently shown that the FXaI microdose cocktail can predict the magnitude of inhibition for the ketoconazole-related exposure changes observed with regular doses of FXaI (Mikus et al., 2019). This study expanded these findings and indicated that the microdose cocktail is also suitable to assess a DDI in a state of induction and in situations with neutral effects.

Although the comparison of dose-normalized exposure showed differences between the microdose and the regular dose, the ${\text{AUC}}_{\infty }$ of the microdose and the therapeutic dose of edoxaban correlated intraindividually at baseline and under induction, suggesting that interindividual differences of the expression and activity of P-gp is likely to influence edoxaban ${\text{AUC}}_{\infty }$ . To highlight intestinal effects, correlation was also tested for C_max_, where only intraindividual correlation between the microdose and the therapeutic dose was found. This confirms previous findings where the ${\text{AUC}}_{\infty }$ of edoxaban did only show intraindividual correlation at baseline but not during inhibition with clarithromycin. (Lenard et al., 2024), which is most likely due to an elimination of variations in the activity of enzymes and transporters, e.g., of P-gp, under the influence of induction (or inhibition as described in Lenard et al. (2024)).

In addition, the baseline ${\text{AUC}}_{\infty }$ of edoxaban as well as the fold ${\text{AUC}}_{\infty }$ decrease during treatment with carbamazepine were significantly correlated. Therefore, the largest reductions of edoxaban exposure by carbamazepine were observed in individuals with the highest baseline exposure and the presumably lowest baseline P-gp activity and/or expression. P-gp modulation affects both, absorption via intestinal extraction, as well as elimination via biliary and renal tubular excretion. These modulations however have been shown to be asymmetric when oral clarithromycin was administered with the paradigm P-gp marker digoxin both oral as well as intravenous. Absorption in the gut was affected the most with only minor changes (15%) in tubular secretion (Rengelshausen et al., 2003; Fenner et al., 2009). Consistent with these results, clarithromycin affected edoxaban asymmetrically when administered as a therapeutic dose as compared to a microdose (Lenard et al., 2024). At the same time t_1/2_ of edoxaban was not significantly changed neither by the perpetrator clarithromycin (Lenard et al., 2024) nor by the perpetrator ketoconazole (Mikus et al., 2019), suggesting that these differences in the extent of DDI are likely transporter-mediated. Our findings are consistent with previous results, where the reciprocal was observed and inhibition was greatest in participants with the lowest ${\text{AUC}}_{\infty }$ of edoxaban at baseline and presumably the highest baseline transporter activity (Lenard et al., 2024). This underlines the assumption that the DDI effect is primarily driven by the perpetrator effect on P-gp. In our trial, the range of exposure changes caused by carbamazepine was 3.1-fold between individuals, ranging from minor exposure decreases to profound reductions (minimum 26% of baseline exposure) and likely loss of anticoagulant activity. For individual patients, the interaction of carbamazepine is therefore far less predicatable than the interaction with the paradigm inducer rifampin, and its consequences appear far more important. Therefore, this combination should be avoided or close monitoring of the anticoagulant effects is desirable if a long-term combination is indicated.

As no specific trials have investigated these drug interactions yet, the exposure changes induced by carbamazepine for microdoses of apixaban and rivaroxaban must be compared with modelling studies or case reports from the literature. For rivaroxaban, recent modelling approaches using a physiologically-based pharmacokinetic model or population pharmacokinetics revealed that carbamazepine is expected to decrease the AUC of rivaroxaban to a range of 0.75 to 0.32 of its baseline values (Ngo et al., 2022; Ngo et al., 2023). This matches well the observed decrease to 0.56 (range 0.38–0.77) in our study. For C_max_ our finding of a reduction to 0.75 was less than suggested by these models (0.64–0.50) (Ngo et al., 2022; Ngo et al., 2023).

For apixaban, there are epidemiological studies and case reports reporting peak and trough concentrations with and without carbamazepine in a clinical setting. The case reports are lacking the predictive statistical power and expectedly show conflicting results, with one case report agreeing well with our findings (Evanger et al., 2017) and the other reporting a considerably smaller interaction (Chadha et al., 2022). Finally, epidemiological studies reported a loss of effect with underdosing of apixaban during carbamazepine (Candeloro et al., 2022). Therefore, all available evidence suggests that a DDI is present and that the individual extent of this DDI is variable and possibly modulated by the baseline activity of P-gp.

### 4.3 Limitations

Although our trial population consisted of healthy volunteers and patients can differ in relevant clinical aspects, the mechanisms of DDI are expected to be similar. Variations in exposure could be at least partially attributed to different release characteristics of edoxaban microdose solution and regular dose, which is a tablet. However, t_max_ after administration of the microdosed solution was rather similar (75 min) to t_max_ of the 60 mg tablet (67 min), indicating that such differences are of minor relevance. Finally, we did not focus on genetic differences because, in an earlier trial, P-gp haplotypes did not predict the extent of clarithromycin-induced exposure changes of rivaroxaban (Gouin-Thibault et al., 2017; Mueck et al., 2013).

## 5 Conclusion

Therapeutic carbamazepine doses reduced the exposure of a therapeutic 60-mg dose of edoxaban to 48% on average with large interindividual variability, which is likely clinically relevant. Carbamazepine also decreased the exposure of microdosed FXaI: apixaban to 66%, microdosed edoxaban to 59%, and microdosed rivaroxaban to 56%. These changes suggest dose adjustments for FXaI and monitoring of FXaI effects to detect loss of effect and to prevent thromboembolic complications, which are always serious events in nature. Therapeutic doses of gabapentin and pregabalin did not change the exposure of a therapeutic 60-mg edoxaban dose nor the exposure of any microdosed FXaI in healthy volunteers, indicating that they can be safely combined without dose adjustment.

## Data Availability

The raw data supporting the conclusion of this article will be made available by the authors, without undue reservation.
