# Supplementary material for: Effect of the frequently used antiepileptic drugs carbamazepine, gabapentin, and pregabalin on the pharmacokinetics of edoxaban and other oral factor xa inhibitors in healthy volunteers
Source: Front Pharmacol. 2025 Apr 11;16:1542063. doi: 10.3389/fphar.2025.1542063 (PMC12022901; doi:10.3389/fphar.2025.1542063)
Supplement: Supplementary file 1 [file DataSheet1.PDF]

**SUPPLEMENTARY MATERIAL to**

# Effect of the frequently used antiepileptic drugs carbamazepine, gabapentin, and pregabalin on the pharmacokinetics of edoxaban and other oral factor Xa inhibitors in healthy volunteers

Alexander Lenard, Simon A Hermann, Felicitas Stoll, Juergen Burhenne, Kathrin I Foerster, David Czock, Gerd Mikus, Andreas Daniel Meid, Walter E Haefeli and Antje Blank

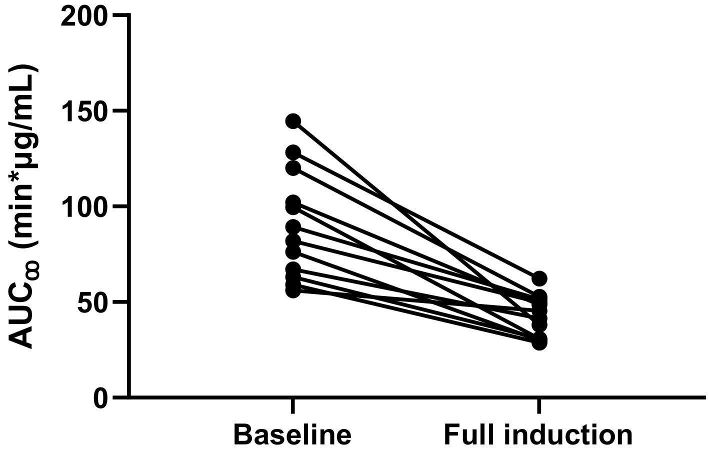


**Supplementary Figure 1** Individual $AUC_{\infty}$ at baseline and at carbamazepine steady-state of a therapeutic dose of edoxaban (60 mg) to 12 healthy volunteers

**Supplementary Table 1** Pharmacokinetic parameters of factor Xa inhibitors at baseline and at carbamazepine steady-state (N = 12 healthy volunteers)

| **Drug (dose)** | **Pharmacokinetic variable** | | **Baseline** | | **At carbamazepine steady-state** | | **P-value of change** |
|---|---|---|---|---|---|---|---|
| | | *Unit* | *GM* | *(95 % CI)* | *GM* | *(95 % CI)* | |
| Edoxaban (60 mg) | CL/F | (L/h) | 42 | (34-51) | 87 | (74-103) | < 0.0001 |
| | $t_{1/2}$ | (min) | 432 | (360-519) | 437 | (328-582) | ns |
| | $t_{max}$ | (min) | 67 | (50-91) | 64 | (45-89) | ns |
| | Vz/F | (L) | 432 | (340-549) | 918 | (642-1313) | 0.003 |
| Edoxaban M-4 | CL/F | (L/h) | 361 | (251-519) | 753 | (555-1022) | < 0.0001 |
| | $t_{1/2}$ | (min) | 288 | (217-383) | 232 | (151-358) | ns |
| | $t_{max}$ | (min) | 133 | (109-162) | 114 | (95.6-135) | ns |
| µ-edoxaban (50 µg) | CL/F | (L/h) | 56 | (48-67) | 96 | (77-119) | < 0.0001 |
| | $t_{1/2}$ | (min) | 298 | (236-376) | 245 | (186-321) | ns |
| | $t_{max}$ | (min) | 75 | (46- 123) | 89 | (53-150) | ns |
| | Vz/F | (L) | 403 | (312-520) | 562 | (421-751) | 0.02 |
| µ-apixaban (25 µg) | CL/F | (L/h) | 4.2 | (3.3-5.4) | 6.4 | (5.2-7.9) | < 0.0001 |
| | $t_{1/2}$ | (min) | 358 | (294-437) | 237 | (200-282) | < 0.001 |
| | $t_{max}$ | (min) | 96 | (67-137) | 103 | (69-154) | ns |
| | Vz/F | (L) | 36 | (27-49) | 36 | (29-45) | ns |
| µ-rivaroxaban (25 µg) | CL/F | (L/h) | 8.0 | (6.2-10) | 14 | (11-18) | < 0.0001 |
| | $t_{1/2}$ | (min) | 314 | (250-394) | 181 | (138-237) | 0.004 |
| | $t_{max}$ | (min) | 49 | (36-65) | 49 | (34-70) | ns |
| | Vz/F | (L) | 61 | (45-82) | 63 | (44-89) | ns |

CI: confidence interval; CL: clearance; F: bioavailability; GM: geometric mean, $t_{max}$: time to reach maximum plasma concentration; $t_{1/2}$: plasma half-life; $V_z$: volume of distribution.

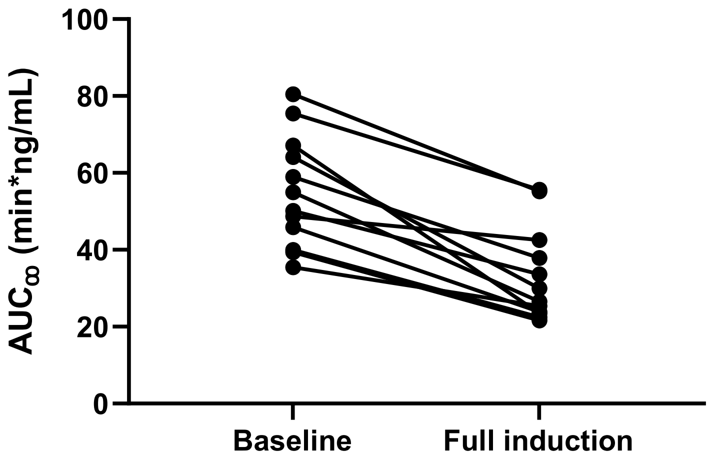


**Supplementary Figure 2** Individual AUC$_\infty$ at baseline and at carbamazepine steady-state after administration of a microdose of edoxaban (50 µg) to 12 healthy volunteers

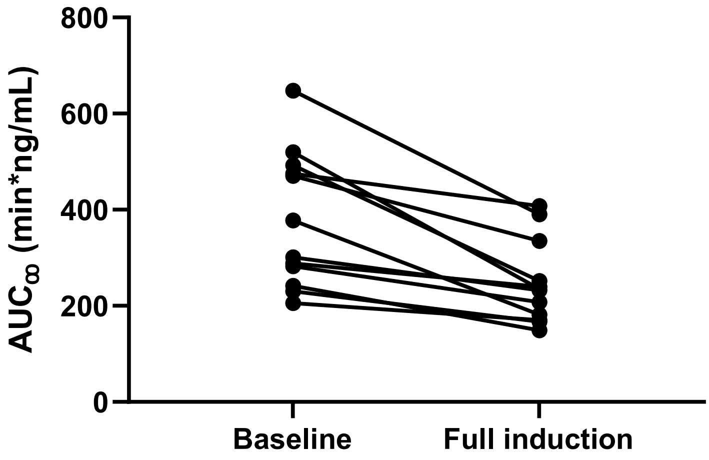


**Supplementary Figure 3** Individual AUC$_\infty$ at baseline and at carbamazepine steady-state after administration of a microdose of apixaban (25 µg) to 12 healthy volunteers

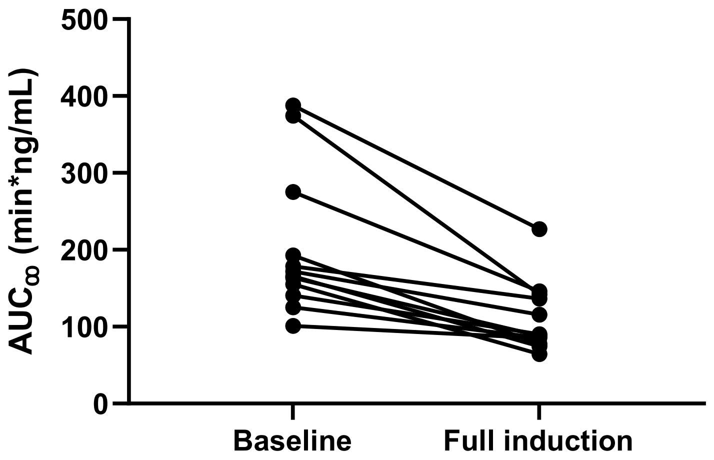


**Supplementary Figure 4** Individual AUC∞ at baseline and at carbamazepine steady-state after administration of a microdose of rivaroxaban (25 µg) to 12 healthy volunteers

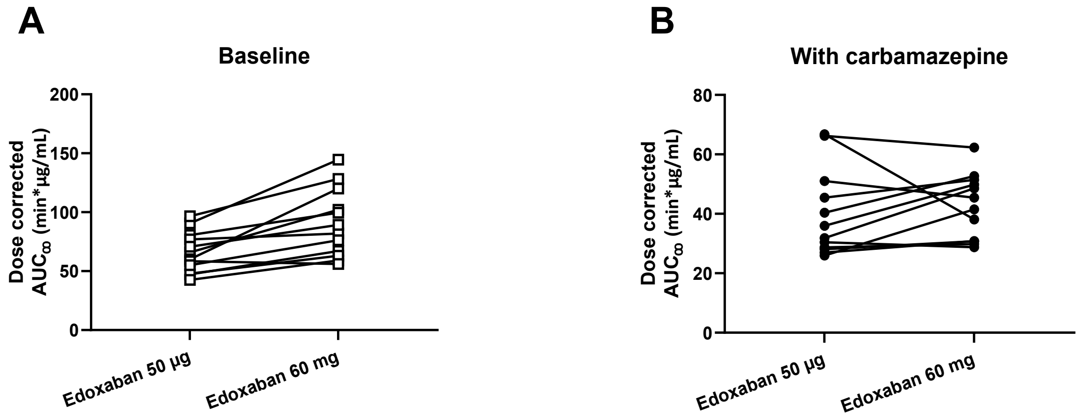


**Supplementary Figure 5** Edoxaban dose-normalized AUC∞ at baseline (A) and during carbamazepine treatment (B) after administration of an edoxaban microdose (50 µg) and a therapeutic dose (60 mg) to 12 healthy volunteers

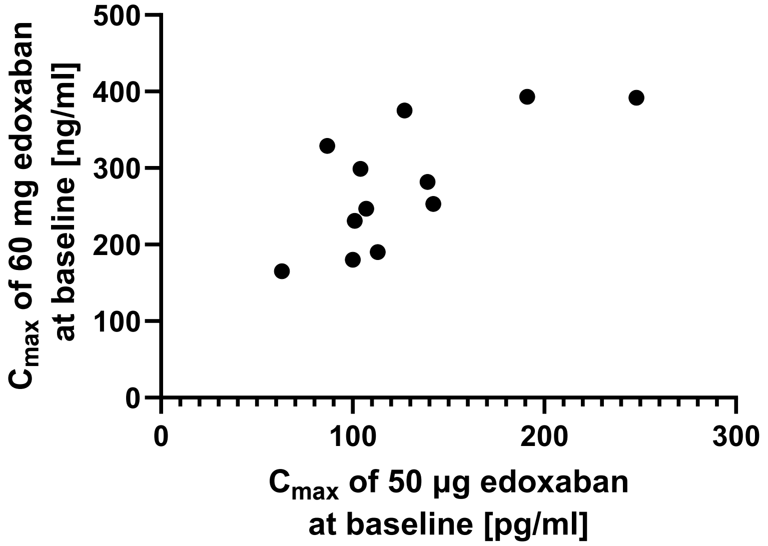


**Supplementary Figure 6** Correlation of maximum plasma concentrations ($C_{max}$) of 60 mg edoxaban and 50 µg edoxaban at baseline

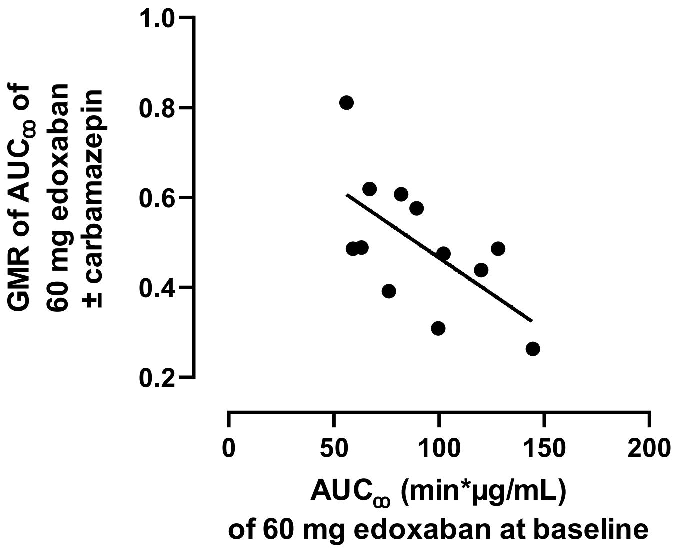


**Supplementary Figure 7** Correlation of individual geometric mean ratios (GMR) of the decrease of the area under the curve ($AUC_{\infty}$) of 60 mg edoxaban at full induction under carbamazepine and $AUC_{\infty}$ at baseline

**Supplementary Table 2** Midazolam pharmacokinetics as a marker for CYP3A activity at baseline and at steady-state of perpetrators (carbamazepine, gabapentin, pregabalin) in 12 healthy volunteers

| **Midazolam**<br>N = 12 | **Baseline** | | **At carbamazepine steady-state** | |
|---|---|---|---|---|
| | **Microdosed FXaI** | **Edoxaban 60 mg** | **Microdosed FXaI** | **Edoxaban 60 mg** |
| $AUC_{2-4}$ (min*ng/mL)<br>(95 % CI) | 3.26<br>(2.75-3.86) | 3.34<br>(2.70-4.13) | 0.59<br>(0.46-0.76) | 0.59<br>(0.45-0.76) |
| *GMR to baseline*<br>*p-value* | | 1.02<br>*ns* | 0.18<br>< 0.0001 | 0.18<br>< 0.0001 |
| $eCL_{met}$ (mL/h)<br>(95 % CI) | 1021<br>(861-1211) | 997<br>(806-1233) | 5636<br>(4349-7303) | 5654<br>(4370-7314) |
| *GMR to baseline*<br>*p-value to baseline* | | 0.98<br>ns | 5.52<br>< 0.0001 | 5.54<br>< 0.0001 |
| **Midazolam**<br>N = 11 | **Baseline** | | **At gabapentin steady-state** | |
| $AUC_{2-4}$ (min*ng/mL)<br>(95 % CI) | 3.55<br>(2.52-5.02) | 3.57<br>(2.42-5.25) | 3.47<br>(2.64-4.57) | 3.60<br>(2.86-4.54) |
| *GMR to baseline*<br>*p-value to base* | | 1.00<br>*ns* | 0.98<br>*ns* | 1.00<br>*ns* |
| $eCL_{met}$ (mL/h)<br>(95 % CI) | 936<br>(662-1324) | 931<br>(632-1373) | 957<br>(727-1260) | 923<br>(732-1163) |
| *GMR to baseline*<br>*p-value to baseline* | | 1.0<br>*ns* | 1.02<br>*ns* | 0.99<br>*ns* |
| **Midazolam**<br>N = 12 | **Baseline** | | **At pregabalin steady-state** | |
| $AUC_{2-4}$ (min*ng/mL)<br>(95 % CI) | 3.13<br>(2.58-3.81) | 2.77<br>(2.30-3.33) | 2.77<br>(2.21-3.48) | 2.54<br>(1.99-3.25) |
| *GMR to baseline*<br>*p-value to baseline* | | 0.88<br>*ns* | 0.89<br>*ns* | 0.81<br>*ns* |
| $eCL_{met}$ (mL/h)<br>(95 % CI) | 1061<br>(873-1289) | 1201<br>(999-1444) | 1198<br>(956-1503) | 1306<br>(1023-1668) |
| *ratio to baseline*<br>*p-value to baseline* | | 1.13<br>*ns* | 1.13<br>*ns* | 1.23<br>*ns* |

$AUC_{2-4}$: area under the concentration-time curve from 2 to 4 h; CI: confidence interval; GM: geometric mean; GMR geometric mean ratio; ns: not significant.

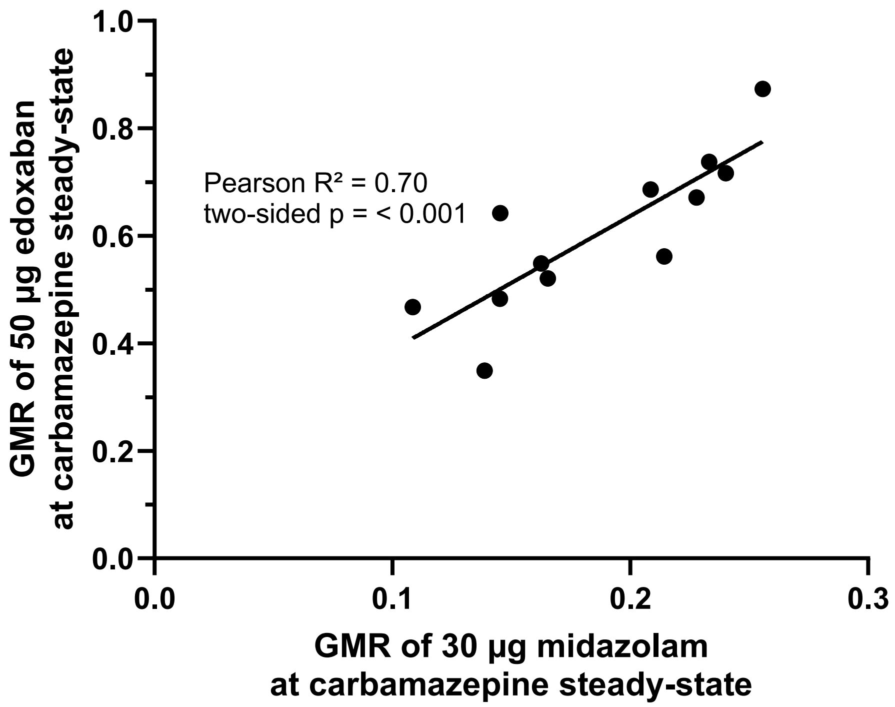


**Supplementary Figure 8** Correlation of geometric mean ration (GMR) of area under the curve ($AUC_{\infty}$) of 50 µg edoxaban and 30 µg midazolam

**Supplementary Table 3** Coagulation parameters measured 180 min after administration of a microdosed factor Xa inhibitor cocktail and after a regular 60-mg dose of edoxaban at baseline and at carbamazepine steady-state

| **Para-meter** | **Baseline** | | **Edoxaban 60 mg alone** | | **Edoxaban 60 mg with carbamazepine** | | **Change at carbamazepine steady-state** | | **p-value** |
|---|---|---|---|---|---|---|---|---|---|
| | *Mean* | *± SD* | *Mean* | *± SD* | *Mean* | *± SD* | *GMR* | *(95 % CI)* | |
| aPTT | 24.78 | 1.25 | 33.63 | 4.71 | 29.56 | 2.44 | 0.96 | (0.95-0.98) | 0.0030 |
| INR | 1.02 | 0.06 | 1.23 | 0.08 | 1.14 | 0.08 | 0.54 | (0.34-0.85) | 0.0012 |
| | | | **FXaI cocktail alone** | | **FXaI cocktail during carbamazepine** | | **Change at carbamazepine steady-state** | | |
| | *Mean* | *± SD* | *Mean* | *± SD* | *Mean* | *± SD* | *GMR* | *(95 % CI)* | |
| aPTT | 24.78 | 1.25 | 24.54 | 1.30 | 24.81 | 1.59 | 1.00 | (0.99-1.01) | ns |
| INR | 1.02 | 0.06 | 1.04 | 0.05 | 1.03 | 0.05 | 1.00 | (0.98-1.01) | ns |

aPTT: activated partial thromboplastin time; FXaI cocktail: combination of 25 µg apixaban, 50 µg edoxaban, and 25 µg rivaroxaban; GMR: geometric mean ratio; INR: international normalized ratio; ns: not significant; SD: standard deviation
